# Complete Nucleotide Sequence of Encephalomyocarditis Virus Isolated from South China Tigers in China

**DOI:** 10.1128/genomeA.00651-13

**Published:** 2013-08-29

**Authors:** Huimin Liu, Qi Yan, Hongxuan He

**Affiliations:** National Research Centre for Wildlife-Borne Diseases, Institute of Zoology, Chinese Academy of Sciences, Beijing, People’s Republic of Chinaa; University of Chinese Academy of Sciences, Beijing, People’s Republic of Chinab

## Abstract

A strain of encephalomyocarditis virus (EMCV), strain FJ13, has been isolated from South China tigers in China, and its complete genome has been sequenced and analyzed. Phylogenetic analysis suggests that FJ13 belongs to the EMCV-1 serotype, and it is highly prevalent in China.

## GENOME ANNOUNCEMENT

Encephalomyocarditis virus (EMCV) (family *Picornaviridae*, genus *Cardiovirus*) is a group of closely related virus strains with a wide host range (1).

EMCV is a positive-sense, single-stranded, nonenveloped RNA virus, with a genome size of ~7,800 bp nucleotides (2). The virus genome contains 5′- and 3′-untranslated regions (UTRs), and a single open reading frame (ORF) encodes a polyprotein that is co- and posttranslationally processed into 12 viral proteins (3). Infection with EMCV is associated with sporadic cases and outbreaks of myocarditis and encephalitis in domestic pigs, numerous species of nonhuman primates, and other mammalian species (4). Since a novel serotype discovery of EMCV-2 in 2012 (5), sequence analyses revealed that EMCV isolates have only two serotypes.

In this study, EMCV strain FJ13 was detected and identified in the heart, liver, and lungs of South China tigers that exhibited clinical signs of myocarditis. Tissue samples were collected and minced, and viral genomic RNA and DNA were extracted from the tissue samples by using TIANamp virus DNA/RNA kit (Tiangen, Beijing, China). The whole ORF of EMCV was amplified and sequenced using a set of specific primers, using the Primer Premier 5.0 software. Both 5′- and 3′-UTRs were amplified by the 5′ and 3′ Full RACE core sets (TaKaRa Biotechnology Company, Dalian, China). The PCR products were cloned into the pMD19-T vector and sequenced by a commercial corporation (BGI, Beijing, China). Sequence assembly was carried out using the SeqMan program of the DNAStar software. The full-genome sequence and assembly of EMCV FJ13 strain generated a sequence of 7,739 bp in length, with a 5′-UTR of 728, a 3′-UTR of 132, and a large ORF of 6,879 nucleotides. Phylogenetic analysis and multiple sequence alignment according to the complete nucleotide sequence revealed that EMCV isolates cluster into two groups (group 1 and 2), with two subclusters within group 1 (group 1a and 1b), and that FJ13 belongs to group 1a.

Comparative genomic analysis showed that FJ13 has 99% nucleotide similarity to the Chinese EMCV isolates NJ08 (GenBank accession no. HM641897), HB1 (accession no. DQ464063), and BJC3 (accession no. DQ464062) from pigs. There was no mutation at amino acids 62 and 231 for VP1 (6) or 156 for VP2. The present paper is the first report of EMCV infection in tigers and provides new epidemiological data on EMCV in China.

### Nucleotide sequence accession number. 

The genome sequence of the FJ13 strain has been submitted to GenBank under the accession no. KF293299.
